# Genome-wide characterization of long intergenic non-coding RNAs (lincRNAs) provides new insight into viral diseases in honey bees *Apis cerana* and *Apis mellifera*

**DOI:** 10.1186/s12864-015-1868-7

**Published:** 2015-09-04

**Authors:** Murukarthick Jayakodi, Je Won Jung, Doori Park, Young-Joon Ahn, Sang-Choon Lee, Sang-Yoon Shin, Chanseok Shin, Tae-Jin Yang, Hyung Wook Kwon

**Affiliations:** Department of Plant Science, Plant Genomics and Breeding Institute, Research Institute of Agriculture and Life Sciences, College of Agriculture and Life Sciences, Seoul National University, Seoul, 151-921 Republic of Korea; WCU Biomodulation Major, Department of Agricultural Biotechnology and Research Institute of Agriculture and Life Sciences, College of Agriculture and Life Sciences, Seoul National University, Seoul, 151–921 Republic of Korea; Department of Agricultural Biotechnology and Research Institute of Agriculture and Life Sciences, College of Agriculture and Life Sciences, Seoul National University, Seoul, 151-921 Republic of Korea

**Keywords:** *Apis cerana*, Asian honey bee, lincRNAs, RNA-seq

## Abstract

**Background:**

Long non-coding RNAs (lncRNAs) are a class of RNAs that do not encode proteins. Recently, lncRNAs have gained special attention for their roles in various biological process and diseases.

**Results:**

In an attempt to identify long intergenic non-coding RNAs (lincRNAs) and their possible involvement in honey bee development and diseases, we analyzed RNA-seq datasets generated from Asian honey bee (*Apis cerana*) and western honey bee (*Apis mellifera*). We identified 2470 lincRNAs with an average length of 1011 bp from *A. cerana* and 1514 lincRNAs with an average length of 790 bp in *A. mellifera*. Comparative analysis revealed that 5 % of the total lincRNAs derived from both species are unique in each species. Our comparative digital gene expression analysis revealed a high degree of tissue-specific expression among the seven major tissues of honey bee, different from mRNA expression patterns. A total of 863 (57 %) and 464 (18 %) lincRNAs showed tissue-dependent expression in *A. mellifera* and *A. cerana*, respectively, most preferentially in ovary and fat body tissues. Importantly, we identified 11 lincRNAs that are specifically regulated upon viral infection in honey bees, and 10 of them appear to play roles during infection with various viruses.

**Conclusions:**

This study provides the first comprehensive set of lincRNAs for honey bees and opens the door to discover lincRNAs associated with biological and hormone signaling pathways as well as various diseases of honey bee.

**Electronic supplementary material:**

The online version of this article (doi:10.1186/s12864-015-1868-7) contains supplementary material, which is available to authorized users.

## Background

Advances in RNA sequencing technologies have allowed the rapid exploration of protein-coding and non-coding RNAs in both vertebrate and invertebrate genomes. Transcriptome sequencing of diverse species has revealed that much of the genome is transcribed. However, only a small portion of sequences code for proteins [1–5]. In human, only less than 2 % of the genome contains evolutionarily-conserved sequences for proteins [3, 6, 7]. Thus, many of the transcribed sequences in the genome are likely to be non-coding RNAs (ncRNAs). Non-coding RNAs include small RNAs [18–35 nucleotides (nt)], as well as longer RNAs (>200 nt) referred as long non-coding RNAs (lncRNAs), for which processing, splicing, and polyadenylation are similar to those of mRNA [8]. Based on their genomic position, lncRNAs can be classified as natural antisense transcripts, long intronic non-coding RNAs, or long intergenic non-coding RNAs (lincRNAs). Recently, lncRNAs have been found to play important roles in biological processes such as the regulation of gene expression through chromatin or histone modification [9–11], or transcriptional [12, 13] and post-transcriptional [14–16] processing. The expression of lncRNAs is often specific to a tissue or a particular developmental stage [17–20]. In addition, lncRNAs are associated with diseases [21] including acquired immune deficiency syndrome (AIDS) [22, 23], Alzheimer’s disease [24], and cancer [25–27], necessitating their study as potential therapeutic targets. Furthermore, lncRNAs including *Xist* play a critical role in X-chromosome dosage compensation [28], genomic imprinting [29], epigenetic regulation [30], cellular pluripotency, and differentiation [31].

It is increasingly clear that lncRNAs are important regulators of diverse functions, and hence, genome-wide scans for lncRNAs are warranted to improve our understanding of cell regulatory and disease-related mechanisms. In recent years, lncRNAs have been studied via EST *in silico* mining [2, 32], whole-genome tilling array, and RNA-seq [32, 33] methods. Genome-wide lncRNA analyses have been performed in human, *Plasmodium falciparum*, mouse, zebrafish, fruit fly, worm, and yeast [34–38]. Each of the surveys in mammals has uncovered a substantial number of lncRNAs, which are often expressed at low levels in a tissue-dependent manner.

Western honey bee (*Apis mellifera*) is a key model for understanding social behavior, disease transmission, and development [39]. The genome of *A. mellifera* was revealed in 2006 [40], which paved the way for understanding regulation of behavior, immunity, and aging, and for molecular and functional genomics studies. A sister species, Asian honey bee (*Apis cerana*), is a significant pollinator in many Asian countries and its genome information was revealed recently, which enables prediction of genes and examination of evolution and comparative sociogenomics between social insects [41]. These two honey bee species have been used in medical research and for studies of neurobiology, developmental biology, behavior science and epigenomics [42–45]. Only four lncRNAs have been characterized in *A. mellifera* [46, 47], and despite their use in clinical research, no effort has been made to profile lncRNAs at the genome level in honey bees.

In the present study, we first generate a comprehensive set of lincRNAs from RNA-seq datasets in *A. cerana* and *A. mellifera.* Secondly, we identify candidate lincRNAs specifically associated with viral diseases in honey bees. Using our bioinformatics pipeline, we identified a total of 2470 lincRNAs, encoded by 2376 gene loci in the *A. cerana* genome (http://mnbldb.snu.ac.kr/; scaffold_v1) and a total of 1514 lincRNAs in the *A. mellifera* genome (www.beebase.org; Amel_4.5_scaffolds), and profiled tissue-specific lincRNA expression. Finally, we characterized the virus-specific lincRNAs in both honey bee species. Our genome-wide profiling of lincRNAs in these two sister honey bee species identifies exciting candidates for characterization of lincRNAs related to diseases as well as to hormone signaling and metabolism, and thus provides valuable information on the modulation of gene expression.

## Results

### Genome-wide identification of lincRNAs from two sister honey bee species, *A. cerana* and *A. mellifera*

**Fig. 1:**
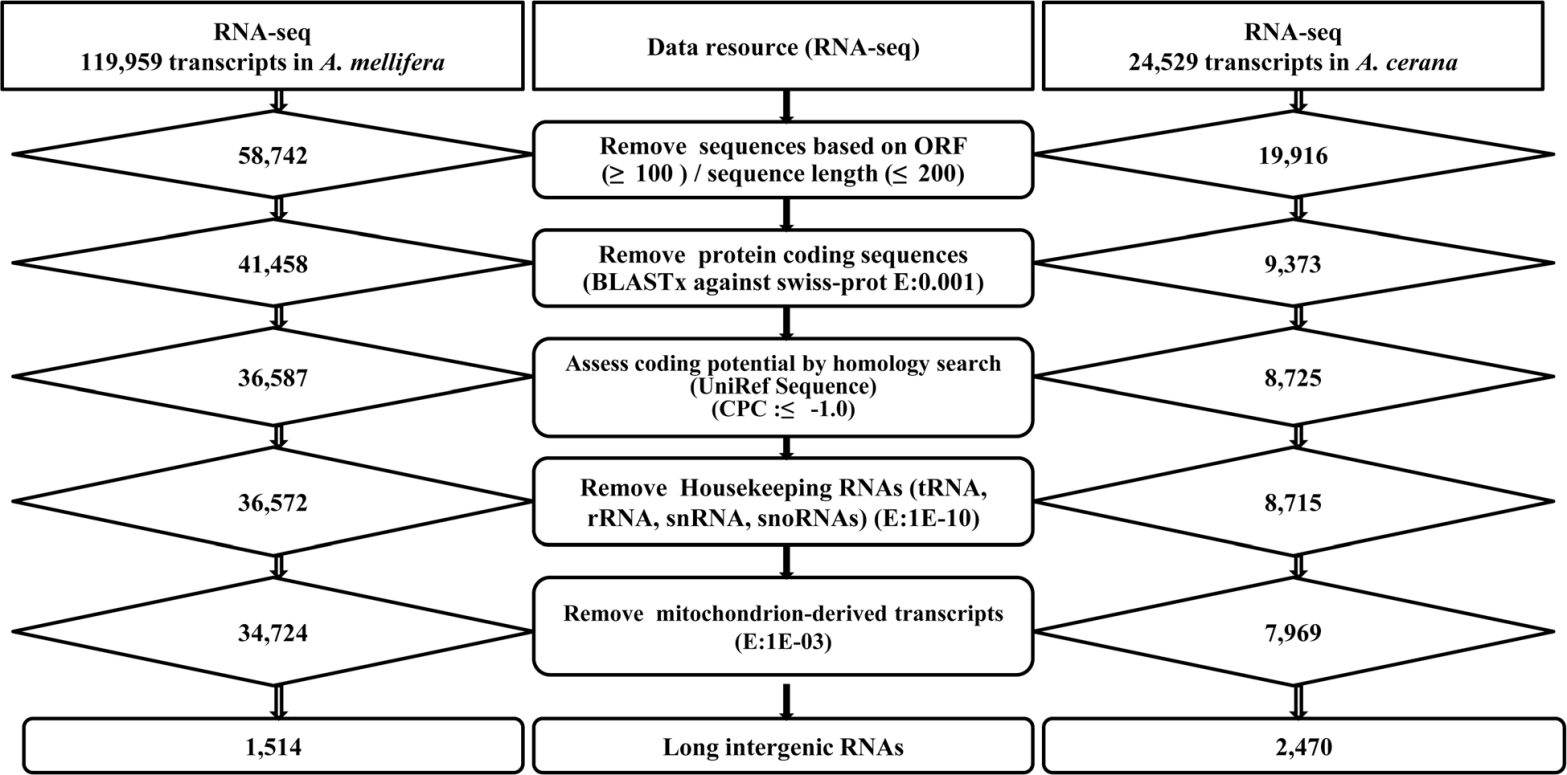
Schematic diagram of the bioinformatics pipeline for lincRNA prediction. Step-wise *in silico* filtration of lincRNAs is shown for both *A. mellifera* and *A. cerana*

To identify a comprehensive set of Asian honey bee lincRNAs, we used Illumina RNA-seq data generated for the *A. cerana* genome project [41] (for six tissues: antenna, brain, hypoharyngeal gland, gut, fat body, and venom gland) and newly generated datasets from larvae, and Sacbrood virus (SBV)-infected and non-infected honey bees (Table 1). We established a bioinformatics pipeline by modifying protocols used in various previous studies [34, 48, 49] (Fig. 1). A reference-guided assembly yielded a total of 24,529 transcripts from 18,937 gene loci. The assembled sequences were analyzed to identify putative lncRNAs, and 19,916 transcripts were selected based on nucleotide length ≥200 bp and ORF ≤ 100 amino acids (Fig. 1). We chose not to consider the protein-coding transcripts in order to increase the accuracy in identifying lncRNAs. From the filtered transcripts, we removed transcripts with overlapping Swiss-Prot protein sequences (http://www.uniprot.org/). The remaining 9373 transcripts were filtered based on coding potential evaluation, removing those with scores ≤ −1.0 using the Coding Potential Calculator (CPC) program, which is a state-of-the-art tool for assessing protein coding potential [50]. It is also necessary to remove pseudogenes and other classes of RNAs such as tRNAs, rRNAs and snRNAs to avoid misprediction. Accordingly, we established a housekeeping RNA database (see Methods) for similarity-based elimination and obtained 8715 putative long non-coding transcripts after removing housekeeping RNAs (Fig. 1). Further, transcripts derived from the mitochondrial genome were filtered by similarity searches against *A. cerana* mitochondrial protein sequences. After applying all these criteria, we identified 7376 candidate loci to encode 7969 putative lncRNAs.

**Table 1:** Details of the RNA-seq data sets from *A. cerana*

Tissue	NCBI SRA	No. of reads	Reference
*A. cerana*			
Antenna	SRR1380976	55,983,150	Park et al. [41]
Brain	SRR1380970	48,168,000	Park et al. [41]
Hypopharyngeal gland	SRR1380979	59,548,970	Park et al. [41]
Gut	SRR1380984	52,489,846	Park et al. [41]
Fat body	SRR1388774	124,626,606	Park et al. [41]
Venom gland	SRR1406762	175,970,162	Park et al. [41]
Larvae	SRR1653580	77,898,496	This study
SBV control	SRR1653605	50,927,126	This study
SBV infected (adult)	SRR1653592	49,363,940	This study

**Fig. 2:**
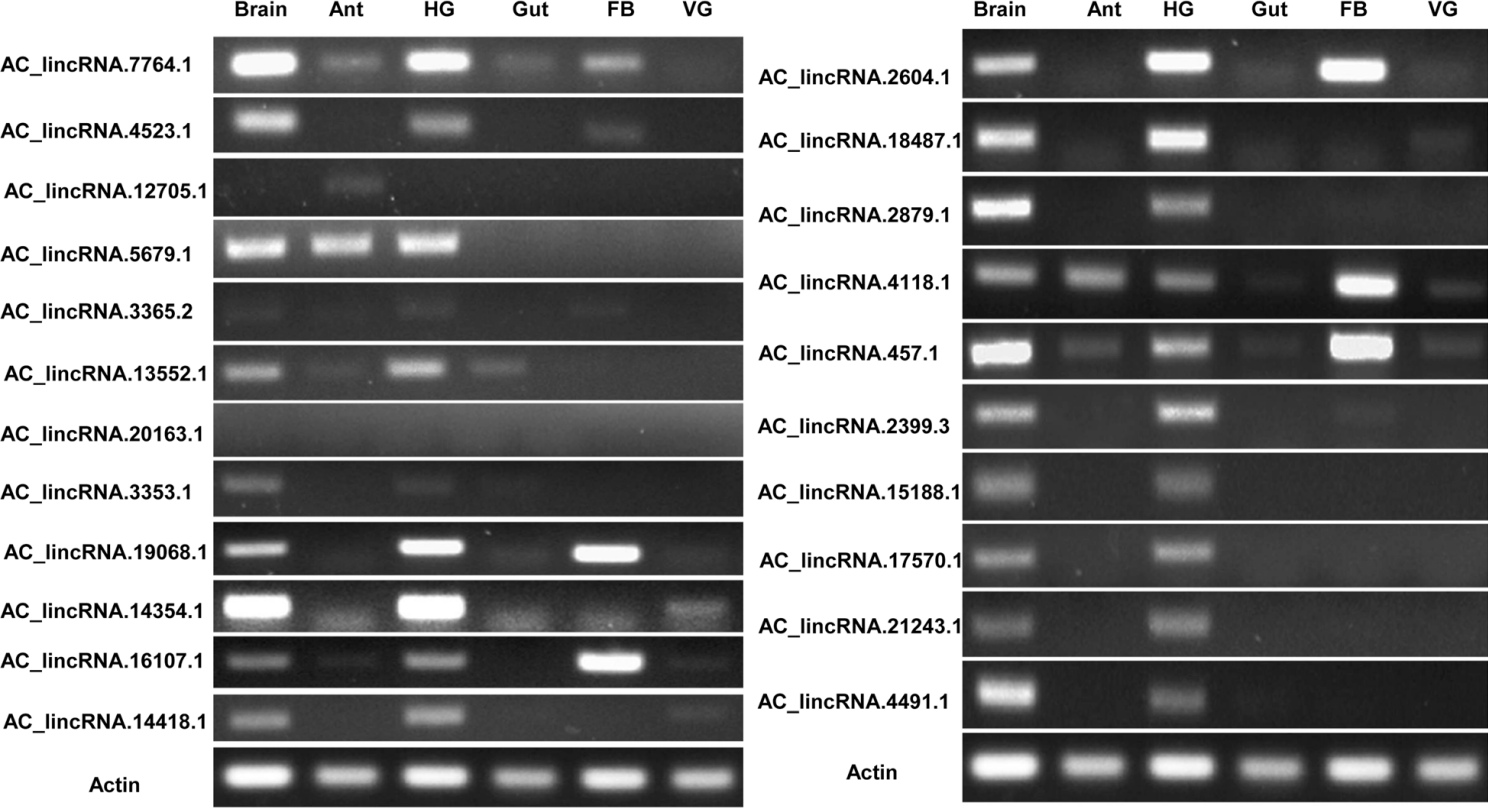
RT-PCR validation of lincRNAs in Asian honey bee (*A. cerana*) tissues. Twenty-two putative lincRNAs were selected for RT-PCR validation. Each primer set was used to perform RT-PCR on six RNA samples, including brain, antenna (Ant), hypoharyngeal gland (HG), gut, Fat body (FB), and venom gland (VG), and *actin* was used as a control to show amplification of cDNA samples

The predicted lncRNAs were further filtered using the *A. cerana* genome annotation [41] to find those that were intergenic, yielding a total of 2470 lincRNAs from 2376 transcription loci (Fig. 1). From these, we selected 22 putative lincRNAs to validate their prediction and expression using RT-PCR. We used six tissues (antenna, brain, hypoharyngeal gland, gut, fat body, venom gland) for RT-PCR confirmation (Fig. 2) in *A. cerana*. The RT-PCR bands and tissue expression patterns were largely consistent with the RNA-seq data (Additional file 1: Table S1 (B). For example, we selected some lincRNAs based on expression in all analyzed tissues (Fig. 2 (Gel: AC_lincRNA.4118.1, AC_lincRNA.457.1) or specifically in 2 (Fig. 2 (Gel: AC_lincRNA.15188.1, AC_lincRNA.21243.1), 3 (Fig. 2 (Gel: AC_lincRNA.4523.1, AC_lincRNA.5679.1), or a single (Fig. 2 (Gel: AC_lincRNA.12705.1, AC_lincRNA.20163.1) tissue. Most of the lincRNAs exhibited tissue-specific expression, and many lincRNA were detected in brain, hypoharyngeal gland and fat body tissues (Fig. 2).

We also identified lincRNAs from *A. mellifera* using the above-validated method (Fig. 1). First, we retrieved a comprehensive set of 119,959 transcripts from the Transcriptome Shotgun Assembly (TSA) database, which was generated from *de novo* assemblies from RNA-seq datasets of seven tissues in the updated genome annotation of *A. mellifera* [39]. This dataset includes separate tissue transcripts from testes (10,054 transcripts), mixed antennae (14,079 transcripts), embryo (18,613 transcripts), brain & ovary (26,425 transcripts), larvae (9107 transcripts), abdomen (14,372 transcripts), and ovary (27,309 transcripts) [39]. Although these transcripts were generated from deep-transcriptome sequencing, no effort has been made to characterize lncRNAs. We obtained 13,775 putative lincRNAs that were not located at introns or overlapping with any protein coding regions in the *A. mellifera* genome according to the latest gene annotations (OGSv3.2). Since these putative lincRNAs were from separate tissue assemblies for each of the seven tissues, it was possible that the same lincRNA transcript was assembled in two or more tissues. Therefore, in order to remove redundancy, we mapped these lincRNAs in the *A. mellifera* genome sequence (Amel_4.5) and found 1514 unique genomic loci where the putative lincRNAs were clustered. LincRNAs clustered at the same locus could not be assumed to be isoforms due to different tissue assemblies, and hence we selected one lincRNA from each locus based on sequence length and consensus alignment with the genome sequence. Ultimately, we obtained a unique set of 1514 lincRNAs in *A. mellifera*, which were used for further characterization. The exon-intron boundaries for each of these lincRNAs were determined based on the genome alignment.

### Characterization of lincRNAs of *A. cerana* and *A. mellifera*

**Fig. 3:**
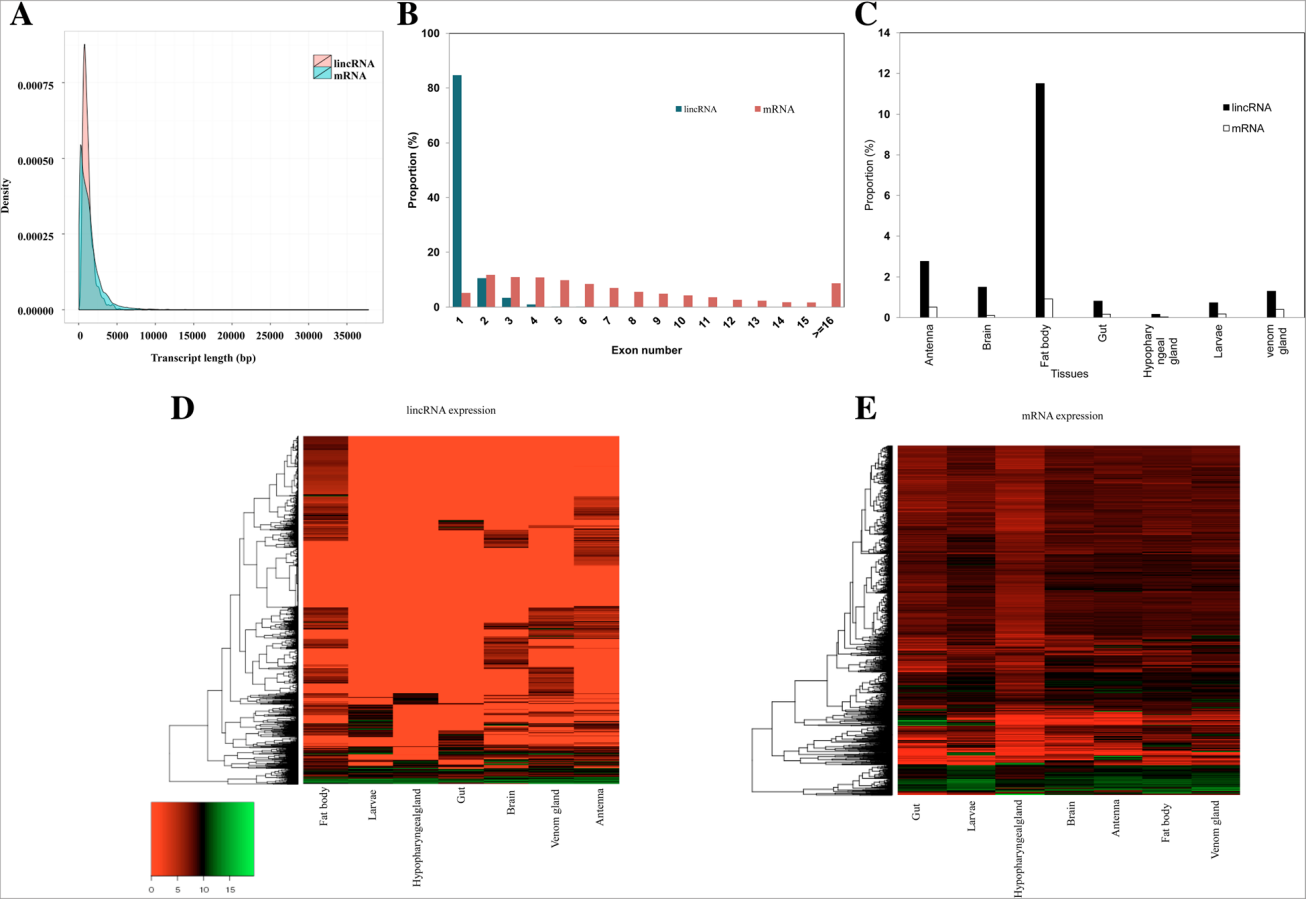
Characteristics of Asian honey bee (*A. cerana*) lincRNAs. **a** Length distribution of lincRNAs and mRNAs. **b** Distribution of exon number in lincRNAs and mRNAs. **c** Percentage of lincRNAs and mRNAs showing tissue-specific expression. **d** Heatmap showing expression levels of all lincRNAs (including the 464 tissue-specific lincRNAs, with a cutoff of a two-fold change identified by EdgeR) and **e** mRNAs based on FPKM in seven tissues (brain, antenna, hypoharyngeal gland, gut, fat body, larvae and venom gland)

To investigate the basic features of *A. cerana* lincRNAs, we compared them with protein-coding mRNAs annotated in the Asian honey bee genome project [41]. Most (84 %) of the lincRNAs consisted of a single exon (Fig. 3b), while mRNAs had exon numbers ranging from 1 to over 16. The average length of lincRNA exons was 1232 bp, which is less than that of protein-coding exons. The majority of lincRNAs (over 85 %) were shorter than 2 kb, with very few (3 %) longer than 3 kb (Fig. 3a). The proportion of lincRNAs ranging from 1 to 2 kb was similar to that of mRNAs. Repetitive analysis was also performed to make a global view of repeat contents in honeybee lncRNAs. We found a low amount of repetitive content in the *A. cerana* lincRNA dataset. We identified as few as 33 retroelements, of which 3 elements were LINEs and 85 were LTR elements. In addition, we identified a total of 16 DNA transposons. On the whole, simple repeats were abundant (2743) compared to the other repetitive elements in lincRNAs. When we aligned the *A. cerana* lincRNAs with those from other species using BLAST with an e-value cutoff of 1E-03, we found detectable sequence similarity to 101 (4.0 %) lincRNAs from *D. melanogaster*, 176 (7.1 %) from *C. elegans*, 65 (2.6 %) from chicken, 217 (8.7 %) from cow, and 144 (5.8 %) from human.

**Fig. 4:**
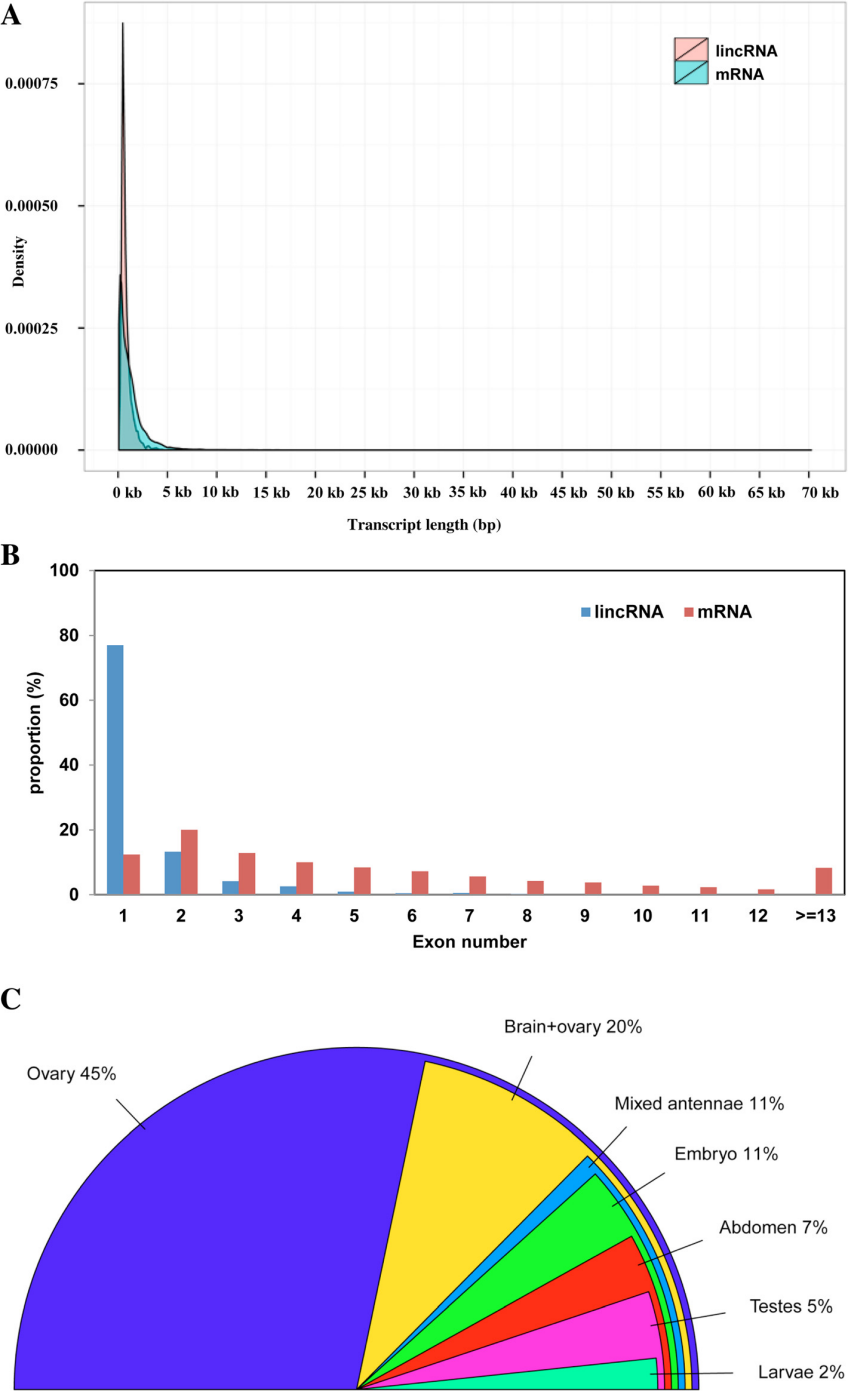
Characteristics of Western honey bee (*A. mellifera)* lincRNAs. **a** Length distribution of lincRNAs and mRNAs. **b** Distribution of exon number in lincRNAs and mRNAs. **c** Percentage of lincRNAs specifically expressed in various *A. mellifera* tissues

Similar results were obtained for *A. mellifera*, in which many of the lincRNAs (77 %) consisted of a single exon (Fig. 4b) and the average length of lincRNAs (790 bp) was shorter than that of annotated protein-coding mRNAs (1266 bp). Similar to *A. cerana*, the majority of the *A. mellifera* lincRNAs were within 2 kb of genes (Fig. 3a), and approximately 18 % were distributed within 400 to 500 bp from a gene. The *A. mellifera* lincRNAs showed similarity to fewer than 2 % of the known lncRNAs from other species and were rich in simple repeats (1156). Annotation files describing the genomic features are available as Additional file 2: Dataset S1 and Additional file 3: Dataset S2; Table 2 shows a comparison of the sequence features of lincRNAs identified in this study. All lincRNAs identified in this study and their respective annotation information can be downloaded at http://mnbldb.snu.ac.kr/data.php.

**Table 2:** Comparison of lincRNAs identified in *A. mellifera* and *A. cerana*

	*A. mellifera* lincRNAs	*A. cerana* lincRNAs
Number of lincRNAs	1514	2470
Total bases	1,197,075	3,044,196
Maximum length (bp)	5248	9150
Minimum length (bp)	200	204
Average length (bp)	790	1232
GC (%)	35	33

### Comparative analysis of lincRNAs between *A. cerana* and *A. mellifera*

To study the evolutionary dynamics of lincRNAs in honey bees, we aligned *A. cerana* lincRNAs to the *A. mellifera* genome and *A. mellifera* lincRNAs to the *A. cerana* genome using BLAST. The vast majority (2360; 95 %) of putative *A. cerana* lincRNAs could be aligned to the *A. mellifera* genome, with identity ranging from 74 to 100 %. Similarly, 1453 (95 %) of *A. mellifera* lincRNAs aligned to the *A. cerana* genome. The remaining un-aligned lincRNAs (5 %) could be specific to each species. Next, we compared lincRNAs from these two sister species with each other and found that 299 *A. cerana* lincRNAs showed perfect matches to 263 *A. mellifera* lincRNAs. The list of 299 lincRNAs in *A. cerana* includes isoforms as identified by Cufflinks and thus includes some sets of multiple transcripts derived from the same locus in *A. cerana* that aligned to a single lincRNA in *A. mellifera*. In addition, we analyzed the exon-intron conservation between these two species based on per-base level identity. The level of identity or conservation was higher for exons as compared to introns in lincRNAs of both species (Additional file 4: Figure S1). Overall, we observed a high level of intron-exon identity (80–100 %) between *A. mellifera* and *A. cerana*, consistent with recent evolutionary divergence.

### Tissue expression profile

Tissue-specific expression is characteristic of lncRNAs and therefore we profiled the expression of lincRNAs in *A. cerana* (Fig. 3d). Sequencing reads from seven tissues were mapped to *A. cerana* lincRNAs separately and the FPKM (Fragments Per Kilobase of exons per Million fragments generated) score was calculated. Many lincRNAs showed low as well as moderate level expression across seven tissues (Fig. 3d). Among them, 22.3 % of the lincRNAs were expressed in at least 2 tissues, 24.6 % were expressed in 3–5 tissues, and 4.6 % were expressed in all seven tissues. Further, differential expression analysis between seven tissues was conducted using edgeR bioconductor package with p ≤ 0.001 and fold change ≥ 2. Overall, we found a subset of 149 differentially-expressed and 464 tissue specifically-expressed lincRNAs. Interestingly, more lincRNAs were preferentially expressed in fat body and antenna tissues than in other tissues in *A. cerana* (Fig. 3c). We also analyzed the analogous characteristics of mRNAs, finding a wide range of expression (low to high FPKM; Fig. 3e). Only 2.3 % of mRNAs showed tissue-specific expression (Fig. 3c) and over 54 % were expressed in all seven tissues.

In *A. mellifera*, the available RNA-seq reads were generated from 400- to 800-bp cDNA fragments by 454 sequencing technology [39]. Generally, long sequencing reads reduce the resolution in gene expression profiling. Therefore, we could not determine the FPKM or RPKM value for *A. mellifera* lincRNAs. However, we determined the expression or presence of each lincRNA in each tissue by comparing each separate tissue assembly with the unique lincRNA set. Approximately 863 (57 %) of the lincRNAs were detected only in one of the seven tissues. Of those, 389 (45 %) and 169 (20 %) were identified in the ovary and brain & ovary tissue assembly dataset, respectively (Fig. 4). Only 3 lincRNAs were identified in all seven tissues, whereas 384 (25 %) and 146 (9 %) lincRNAs were expressed in two and three tissues, respectively.

### Candidate lincRNAs associated with honey bee viral diseases

One major aim of this study was to identify candidate lincRNAs associated with honey bee diseases. SBV disease is a major diseases of honey bee, especially *A. cerana*, in which the SBV attacks brood and adult stages of bees and thus leads to decreased life span [51]. We compared lincRNA expression between SBV-infected and non-infected (control) honey bees. First, we analyzed the RNA-seq data set derived from the SBV control and infected bees of *A. cerana*. We identified 15 lincRNAs that showed significant differential expression between SBV control and infected data using read mapping and the edgeR bioconductor package. We selected those differentially-expressed transcripts for qRT-PCR validation in both adult and larvae stages of *A. cerana*. Of 15 lincRNAs, 11 showed expression consistent with our RNA-seq results of significant differential expression between SBV control and infected honey bees. Among them, one lincRNA (ID: AC_lincRNA.3472.1) was down-regulated and the rest were up-regulated in both adult and larval SBV-infected *A. cerana*. These lincRNAs represent candidates to play specific roles in SBV replication and regulation of SBV-resistance genes.

**Fig. 5:**
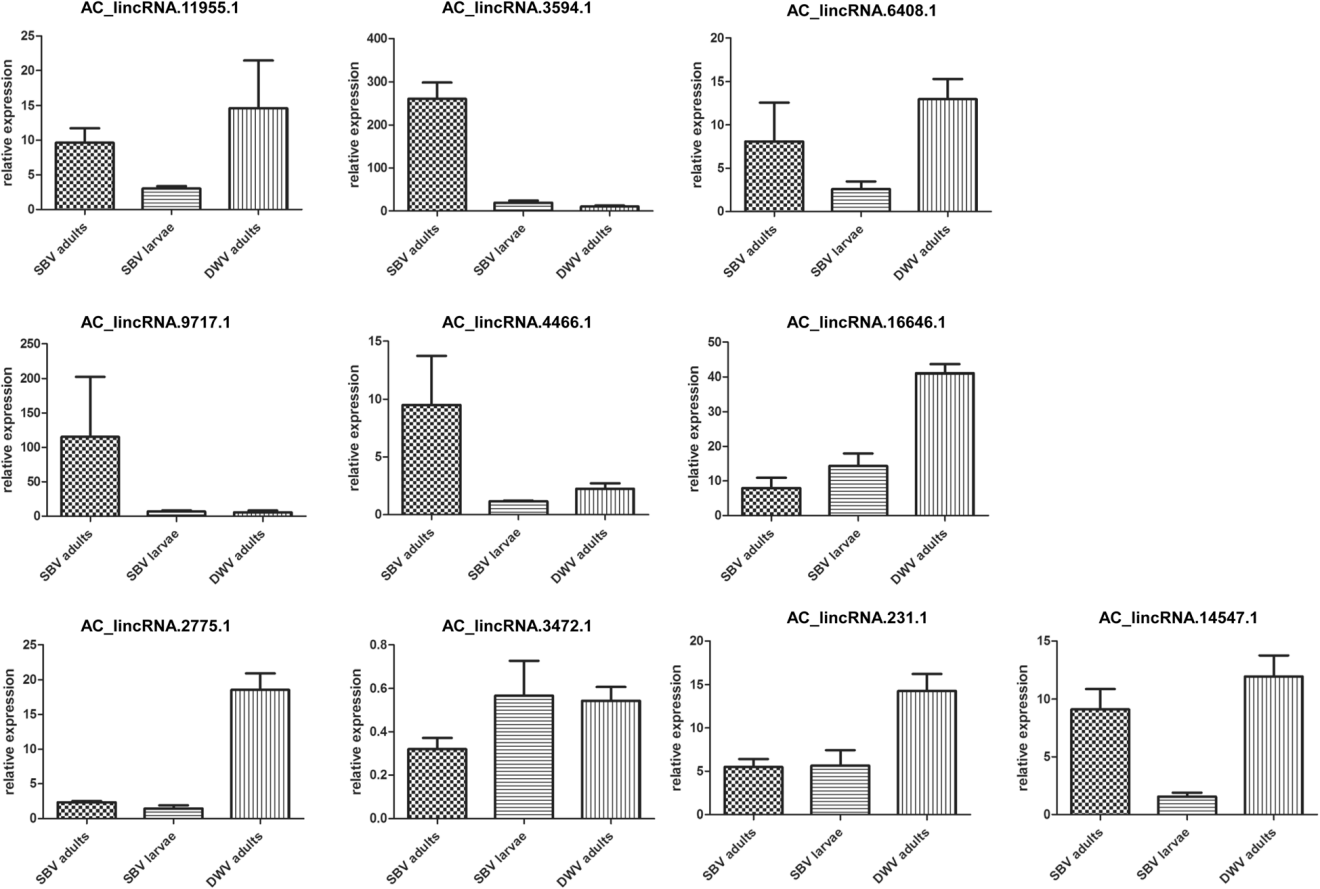
Differential expression of virus-specific lincRNAs. Relative expression of lincRNAs between uninfected (Control) and SBV- and DWV-infected bees. Quantitative analysis was carried out using StepOne plus Software V. 2.0 (Applied Biosystems). Results were normalized to a validated control gene, *actin* (data not shown), for which values were set to 1, using the ΔΔCt method for each sample [58]. SBV- and DWV-infected samples were collected from *A. cerana* and *A. mellifera*, respectively

Additionally, we examined the responses of these lincRNAs to other viral diseases in the honey bee to investigate if they are specific to SBV. DWV, a viral disease closely linked to *Varroa* mite infestation [52], causes wing deformity and premature death in adult honey bees of *A. mellifera* [52]. Hence, we investigated the expression patterns of those same 11 lincRNAs in DWV-infected and healthy uninfected *A. mellifera* honey bees using qRT-PCR*.* Intriguingly, we found that 10 of the lincRNAs showed a similar expression pattern in response to infection in this species, including the one down-regulated (AC_lincRNA.3472.1; Fig. 5). Furthermore, RT-PCR products for 10 lincRNAs from *A. cerana* were sequenced and found to match exactly to those lincRNAs (Additional file 5: Dataset S3). Together, these results suggest that this subset of 10 lincRNAs may play critical roles in pathogen-host interactions in honey bees. Therefore we regarded these lincRNAs as virus-specific lincRNAs in honey bee. We have submitted these virus-specific lincRNAs to GenBank (Acc. Nos.: KM889914-KM889923).

Most of these virus-specific lincRNAs contain a single exon, except AC_lincRNA.16646.1 and AC_lincRNA.3472.1, which contain two exons. The size of these lincRNAs ranged from 322 to 813 bp. In terms of digital expression, apart from the SBV datasets, AC_lincRNA.16646.1 and AC_lincRNA.4466.1 were found to have low-level expression in healthy non-infected antenna, brain, fat body and venom gland tissues, whereas AC_lincRNA.2775.1 and AC_lincRNA.231.1 showed expression only in fat body. AC_lincRNA.14547.1 and AC_lincRNA.3472.1 were uniquely found in the brain and venom gland datasets, respectively. The remaining lincRNAs were identified only in the SBV dataset. By contrast, although these lincRNAs identified in *A. cerana* were found in the genome sequence of *A. mellifera*, they were not identified in any of the *A. mellifera* tissue transcriptomes, which were assembled from healthy honey bees, suggesting that these lincRNAs were not expressed or were expressed at too low a level to be detected by RNA sequence data. These findings support the idea that the set of virus-specific lincRNAs are expressed only upon viral infection in both honey bee species. Using BLAST, we found that the level of identity of these candidate lincRNAs with those from *A. mellifera* ranged from 91 to 96 % both in exon and intron regions. This high level of identify suggests that the set of shared candidate lincRNAs might function similarly in the two species.

## Discussion

RNA sequencing (RNA-seq) is a powerful tool that enables the research community to discriminate cellular transcripts quantitatively [53]. It has been successfully employed for transcriptome profiling in various model and non-model organisms [54, 55]. Similarly, RNA-Seq has been used for lncRNA identification in both plants and mammals [56–60]. Due to numerous potential roles of lncRNAs in the genome and in organismal development [9–16], characterizing lncRNAs has become essential to understand gene regulation in eukaryotic species.

### Identification of lincRNAs in honey bees

Previously, four lncRNAs, two from the adult brain [47] and two from the larval ovaries [46], were identified in *A. mellifera*, all of which were intronic and natural anti-sense type. In this study, we have identified a relatively robust set of potential lincRNAs from *A. cerana* (Asian honey bee), which is a good model for behavior research due to their fascinating habits of grooming, hygiene and aggregation against predators [41], and *A. mellifera* (Western honey bee) which has served as a key model for social insects [40]. We used a total of 694 million sequencing reads from *A. cerana* and identified a set of lincRNAs using our bioinformatics protocol. In general, transcriptome assembly produces many partial sequences and some antisense transcripts that can be aligned only at intronic regions of a protein coding gene rather than overlapping onto that gene’s exons due to partial assembly. It is possible that such transcripts could be wrongly annotated as intronic-type lncRNAs as they might not contain potential ORFs or protein-coding domains. Therefore, intergenic type lncRNAs are most appropriately and reliably predicted from RNA-seq datasets obtained using poly-A primers and non-strand-specific methods, and hence, our lincRNA datasets did not include non-polyadenylated, antisense, nor intronic-type lncRNAs. In our current data, the distance between identified lincRNAs and UTR regions of the neighboring genes varies from over 200 bp to 1 kb. It is also possible that some of the lincRNAs, for which we might have only partial data at present, span a UTR region, especially if the honeybee genomes have poor UTR annotation. This possibility can be addressed in the future with more comprehensive transcriptome assembly and genome annotation from very large-scale RNA-seq data. We have made available the lincRNA features in GTF file format, which can be used in genome browsers and should enhance the honey bee genome annotation. LncRNAs show more plasticity than protein-coding genes and thus lack of conservation in general [61, 62]. Not surprisingly, our lincRNAs also exhibited rather less similarity to those of other species and shared more similarity with those of the sister species.

### Expression profiling

Similar to the results of other lncRNA studies, we identified a subset of tissue-dependent lincRNAs with almost no expression elsewhere, suggesting that the expression of these lincRNAs is tightly controlled in a tissue/development-specific manner. Approximately 11 % of the total lincRNAs were uniquely expressed in fat body tissue in *A. cerana*. Fat bodies, in which energy is stored and released according to the demands of the insect, play an important role in honey bee health [63]. In addition, major metabolic and hormone signaling pathways reside in fat bodies [64]. Consistent with our results, lncRNAs have been implicated in the development of insulin resistance and tissue-specific regulation of metabolism [65]. Therefore, we speculate that lincRNAs in fat bodies may be involved in various metabolic pathways, including hormone biosynthesis, in *A. cerana*. Ovary has also been reported as a preferential tissue for lncRNA expression [66], and we identified many lincRNAs specific to *A. mellifera* ovary tissue. We found more tissue-specific lincRNAs in *A. mellifera* than in *A. cerana*, which could be due to unequal sequencing depth and biased transcriptome assembly in the various tissues.

### Implications for disease-specific lincRNAs of the honey bee

To date, 22 viruses have been reported to infect honey bees [52], and many of these have also been reported to be associated with *Varroa* parasitism [52]. Pathogens are proposed to be the major contributors to honey bee mortality [52]. Previously, Peng et al. [67] discovered that lncRNAs exhibit unique expression in response to viral infection [68] in mice. Here, we identified candidate lincRNAs associated with viral diseases of honey bee. Among the virus-specific lincRNAs, one lincRNA was expressed more highly in healthy adult bees of both *A. mellifera* and *A. cerana*, whereas the ten others were all up-regulated in both SBV- and DWV-infected bees. This demonstrates that the virus infections modulate the expression levels of many lincRNAs. Since we identified 11 lincRNAs as differentially expressed in SBV-infected honey bee, our findings support the idea that lncRNAs can be regulators in determining the outcome of infection as demonstrated by Peng et al. [66]. Ten lincRNAs were also observed as responding to two different viral diseases, suggesting that a subset of lncRNAs plays critical roles during viral infection in general. Determining what specific roles lincRNAs play in virus-host interactions awaits further research.

## Conclusion

Emerging reports have suggested that lncRNAs play important functional roles in disease, development and various biological processes in eukaryotes. In this study, we have provided a comprehensive set of lincRNAs in honey bees. We identified more than 1000 lincRNAs in *A. cerana* and *A. mellifera*, which were likely to exhibit tissue-specific expression patterns, as validated by expression profiling and RT-PCR analysis. The lincRNAs were less conserved than protein-coding mRNAs and contained low repeat content. Finally, we identified lincRNAs associated with SBV and DWV diseases and confirmed their differential expression by qRT-PCR. This study thus provides the first comprehensive genome-wide analysis of honey bee lincRNAs and paves the way for identification of lincRNAs associated with general development, biological and hormone signaling pathways and disease resistance.

## Methods

### RNA isolation and next generation sequencing

Bees of *A. cerana* were taken from 3 different colonies at an apiary located at the College of Agriculture and Life Sciences, Seoul National University (SNU), Seoul, Korea during the summer season. While we conducted this experiment, the queen bee was not changed genetically. Worker bees were captured and directly placed in liquid N_2_, and stored at −80 °C. Tissues were dissected in cold RNase-free PBS (pH = 7.4). Total RNA was isolated from *A. cerana* larvae and SBV-infected and non-infected honey bees using a QIAGEN RNeasy Mini kit (Qiagen, CA, US) according to the manufacturer’s protocol. The complementary DNA was prepared for each tissue using the Illumina mRNA sequencing kit (Illumina, CA, US) and the Clontech SMART cDNA Library Construction Kit (Invitrogen). Libraries were sequenced using Illumina HiSeq2000.

### Pipeline for identifying lincRNAs

1) RNA-seq data were aligned to the *A. cerana* reference genome using the spliced aligner Tophat [46] (with --no-discordant, −-no-mixed parameters) and then assembled using Cufflinks [47] (with parameters -u --library-type fr-unstranded).

2) The assembled transcripts were initially filtered based on size and ORF. An in-house perl script was used to select transcripts that were ≥200 bp in length and ORFs of ≤100 amino acids.

3) Then, transcripts were compared to the Swiss-Prot protein database to eliminate protein coding transcripts using BlastX with an E-value cutoff of 1E-03.

4) To calculate the coding potential, the coding potential calculator (CPC) [48] was utilized with default parameters. Transcripts with non-coding scores were considered as lncRNAs.

5) To eliminate housekeeping non-coding RNAs (transfer (t) RNAs, small nuclear (sn) RNAs and small nucleolar (sno) RNAs), a housekeeping RNA database was made, and putative lncRNAs were aligned to the database with an E-value cutoff of 1E-10. This database contained tRNA sequences from the genomic tRNA database (http://gtrnadb.ucsc.edu/), rRNAs from the silva database (http://www.arb-silva.de/no_cache/download/archive/current/Exports/), and other ncRNAs (snRNAs, snoRNAs, 7SL/SRP) downloaded from NONCODE (http://noncode.org/).

6) Transcripts derived from the mitochondrial genome were removed by alignment against the mitochondrial protein sequences of *A. cerana* and *A. mellifera* downloaded from NCBI (GenBank accession GQ162109 and NC_001566).

7) Finally, gene annotation information for both *A. cerana* (http://mnbldb.snu.ac.kr/) [41] and *A. mellifera* (OGSv3.2: http://hymenopteragenome.org/beebase/) were retrieved from their genome databases. An in-house python script was developed to identify lncRNAs located between two genes, and those lncRNAs were regarded as lincRNAs in this study.

### Expression and alignment of lincRNAs

Exon-intron alignment between the two honey bee sister species were performed using BLAST with over 40 % alignments and an E-value threshold of e^−10^. Tissue specificity was determined based on the condition that the FPKM should be >1 in the specific tissue and zero in the rest of the tissues. This condition was set based RT-PCR results from randomly selected lincRNAs in *A. cerana*.

### Validation of putative lincRNAs by RT-PCR

RT-PCR was conducted for 22 lincRNAs in 6 tissues (antenna, brain, hypoharyngeal gland, gut, fat body, venom gland) in *A. cerana*. The primer pairs were selected using Primer 3, and the primer sequences are presented in Additional file 1: Table S1 (A). PCR was conducted using 2X Premix-MG Taq (Macrogen, Cat No. MP018S) following the manufacturer’s instructions under the following conditions: pre-denaturation step at 95 °C for 3 min; 30 amplification cycles of denaturation at 95 °C for 30 s, annealing at 50 °C for 30 s, and elongation at 72 °C for 30 s; followed by a final elongation step at 72 °C for 5 min. Electrophoresis was conducted using 1.2 % agarose gels.

### Quantitative real-time PCR assay

Expression of selected differentially expressed lincRNAs between SBV control and infected bee samples was analyzed through qRT-PCR. RNA was isolated from the SBV-infected and healthy adult as well as larval bees of *A. cerana* using the RNeasy kit (Qiagen) according to manufacturer’s instructions. Similarly, RNA was isolated from DWV control and infected bees of *A. mellifera*. cDNA was prepared from 500 ng RNA using Superscript III (Invitrogen, USA). The PCR amplification was carried out using SYBR Green PCR Master Mix (Applied biosystems, UK). Primer sequences are presented in Additional file 6: Table S2. The data presented here represent three independent biological and technical replicates.

### Availability of supporting data

The RNA-seq data generated in this study are available in the NCBI Sequence Read Archives (SRA) with accessions SRR1653580, SRR1653605, and SRR1653592 for larvae, SBV-non-infected (control), and SBV-infected adult bees of *A. cerana*. The candidate virus-specific lincRNAs are available at GenBank (KM889914-KM889923). All the assembled lincRNAs and their analysis results are available at http://mnbldb.snu.ac.kr/data.php.

## Additional files

Additional file 1: Table S1. (A) Primers used for RT-PCR validation and (B) FPKM values of seven tissues of *A. cerana* used in this study. (XLS 328 kb) Additional file 2: Dataset S1. Annotation of *A. cerana* lincRNAs. (GTF 1130 kb) Additional file 3: Dataset S2. Annotation of *A. mellifera* lincRNAs. (GTF 403 kb) Additional file 4: Figure S1. Intron-exon conservation of lincRNAs between *A. mellifera* and *A. ceana*. Y-axis (density) represents the total lincRNAs and X-axis indicates their identity. (TIFF 1637 kb) Additional file 5: Dataset S3. Sequenced PCR products of virus-specific lincRNAs. (TXT 1 kb) Additional file 6: Table S2. Primers used in qRT-PCR analysis. (XLS 28 kb)

## Abbreviations

lincRNA: Long intergenic non-coding RNA
SBV: Sacbrood virus
DWV: Deformed wing virus
lncRNA: Long non-coding RNA
bp: Basepair
kb: Kilobase
FPKM: Fragments per kilobase of exon per million fragments
PCR: Polymerase chain reaction
OGS: Official gene set
ORF: Open reading frame
