# Supplementary figures and images for: Genome-wide characterization of long intergenic non-coding RNAs (lincRNAs) provides new insight into viral diseases in honey bees Apis cerana and Apis mellifera
Source: BMC Genomics. 2015 Sep 4;16(1):680. doi: 10.1186/s12864-015-1868-7 (PMC4559890; doi:10.1186/s12864-015-1868-7)

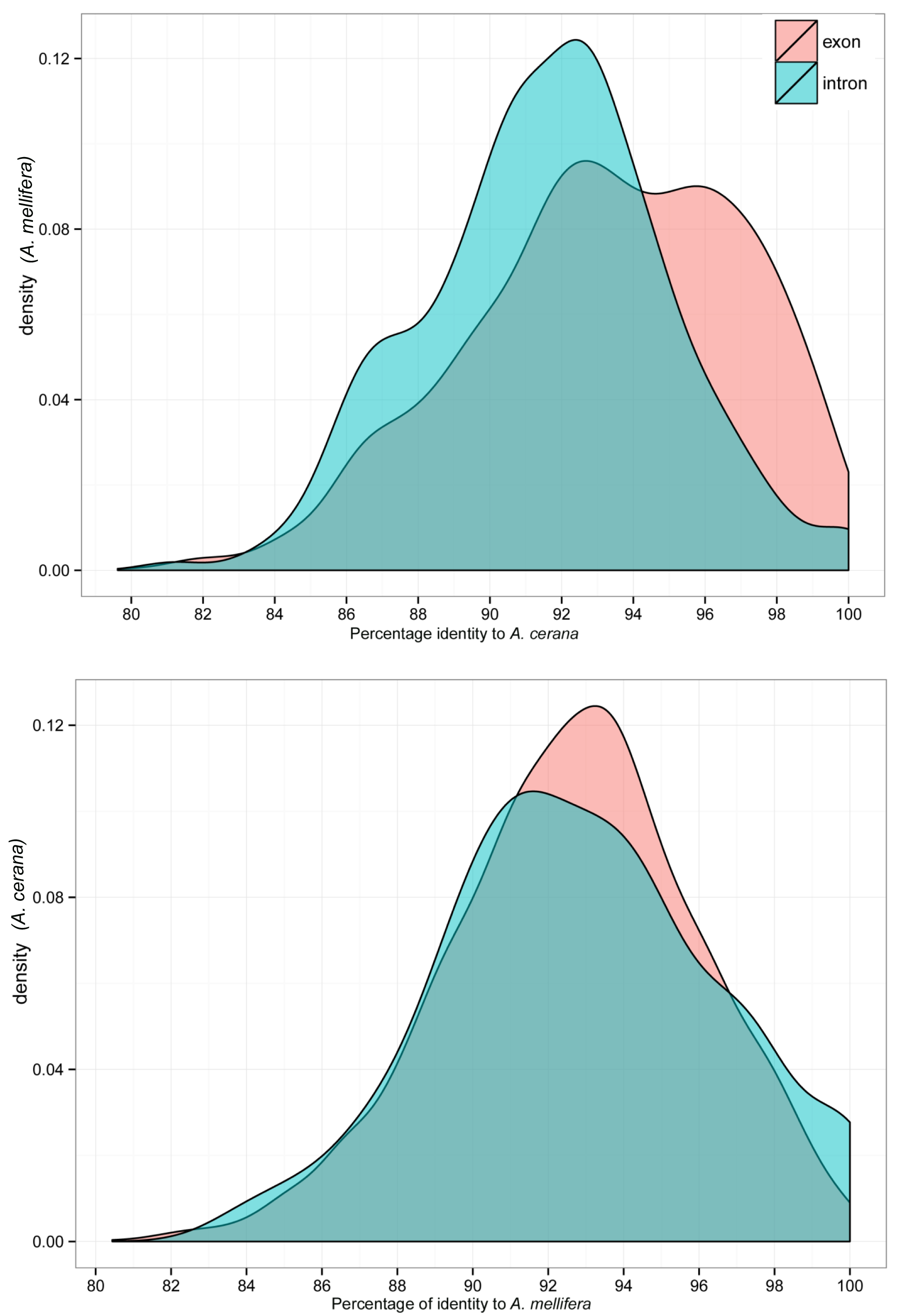

Supplement: Additional file 4: Figure S1. — Intron-exon conservation of lincRNAs between A. mellifera and A. ceana. Y-axis (density) represents the total lincRNAs and X-axis indicates their identity. (TIFF 1637 kb) [file 12864_2015_1868_MOESM4_ESM.tiff]
